# Isolation and Characterization of β-Phenylethylamine-Producing Lactic Acid Bacteria from Dairy Products

**DOI:** 10.3390/microorganisms13050966

**Published:** 2025-04-23

**Authors:** Angel Casado, Eva Fernández, Héctor González, María Fernández, Miguel A. Alvarez, Victor Ladero

**Affiliations:** 1 Department of Technology and Biotechnology of Dairy Products, Dairy Research Institute, IPLA-CSIC, C/Francisco Pintado Fe 26, 33011 Oviedo, Asturias, Spain; angel.casado@ipla.csic.es (A.C.); eva.fernandez@ipla.csic.es (E.F.); hectorgi@ipla.csic.es (H.G.); mfernandez@ipla.csic.es (M.F.); maag@ipla.csic.es (M.A.A.); 2 Instituto de Investigación Sanitaria del Principado de Asturias (ISPA), Av. del Hospital Universitario s/n, 33011 Oviedo, Asturias, Spain

**Keywords:** neuroactive compounds, β-phenylethylamine, *Enterococcus durans*, dairy, tyrosine decarboxylase, gene knockout

## Abstract

β-phenylethylamine (PEA) is a neuroactive trace amine synthesized by the enzymatic decarboxylation of phenylalanine. PEA is involved in the improvement of mood and attention. Functional foods enriched in this compound could, therefore, be of interest to the food industry. PEA is produced by microbial activity in certain foods, but usually only in small amounts. The search for PEA-producing microorganisms with good technological properties is thus a pre-requisite if such functional foods are to be produced. This work reports the isolation of thirty-three PEA-producing bacterial strains from samples of different dairy products. They belong to the genus *Enterococcus*, and the species *Levilactobacillus brevis*. Identified strains of *Enterococcus durans* were then selected for technological characterization. Some of them showed properties of interest. In this species, PEA was determined to be produced via the action of tyrosine decarboxylase, encoded by the gene *tdcA.* This implies that, apart from PEA, a concomitant production of tyramine, a toxic biogenic amine, was observed.

## 1. Introduction

β-phenylethylamine (PEA), the decarboxylation product of phenylalanine, is an aromatic, cyclic, biogenic amine (BA) that belongs to the trace amines—a family of endogenous amines related to classical monoamine neurotransmitters present in all invertebrate and vertebrate species [1,2,3] where it functions as an endogenous neuromodulator of central monoaminergic neurotransmission, typically providing a stimulant signal [4]. It also alters monoamine transporter function, promoting an increase in the concentration of neurotransmitters, such as dopamine, serotonin, and norepinephrine, all of which have been associated with improvements in mood, physical energy, and attention [5,6,7]. Altered levels of PEA are reported to be involved in neurological and psychiatric diseases, including depression, attention deficit hyperactivity disorder, and schizophrenia [8,9,10]. Worldwide, depression and related mental disorders are estimated to affect more than 500 million people, and the number is growing [11]. Patients with depression are usually treated with antidepressants, especially selective serotonin reuptake inhibitors (SSRI). These work by blocking the serotonin transporter, thereby inhibiting the reuptake of serotonin and increasing its synaptic concentration [12]. Since PEA alters the serotonin transporter by interacting with trace amine-associated receptors (TAARs), it has been suggested as a safer and effective alternative to SSRIs [4,13]. Moreover, it is gaining popularity as a nutritional supplement for improving mood, well-being, and attention, as well as for helping with weight loss and athletic performance [14]. 

The connection between “food and mood” has long been recognized [15,16], with chocolate being a focus of intense research in this respect [17,18]. As well as containing beneficial substances, such as antioxidants [18], chocolate also contains PEA [19], perhaps investing it with its anti-stress effects and mood improvement properties [18]. Certainly, it has been shown that healthy dietary patterns can effectively reduce depressive symptoms and improve cognitive ability, suggesting that dietary interventions [20], including the use of food supplements [21], might be useful in preventing or treating depression [22,23].

PEA has also been found in fermented foods, especially sausages and fish derivatives (up to 182 mg kg^−1^) [24], and in dairy products. Cheese may contain high concentrations of different BAs, including trace amines, a consequence of the favourable conditions it provides for the decarboxylating reactions involved in the synthesis of these compounds [25,26]. It is well known that the ingestion of cheese with high concentrations of BAs can cause physiological changes [25], suggesting that it might be a good candidate as a PEA-containing functional food. The concentration of PEA in cheese is generally reported to be low [27,28], although some authors have detected over 400 mg kg^−1^ in certain cheese types [29,30].

The accumulation of PEA in fermented foods is the result of the microbial decarboxylation of the amino acid phenylalanine [31]. Although the ability of different foodborne bacteria, including some lactic acid bacteria species such as *Levilactobacillus brevis*, *Lactilactobacillus curvatus*, and some enterococci to produce PEA has been described [31,32], no specific phenylalanine decarboxylase enzymes have ever been found. In fact, the ability of some bacteria to produce PEA rests on the action of tyrosine decarboxylase (TDC), for which phenylalanine is an alternative substrate [33]. In dairy products, the report of foodborne strains to produce PEA is scarce; only a few strains belonging to *Enterococcus faecalis*, *Enterococcus faecium,* and *Enterococcus durans* have been described with the capacity to decarboxylate phenylalanine and produce PEA [34,35]. However, both *E. faecalis* and *E. faecium* have been implicated in nosocomial infections, and the fact that they frequently carry virulence factors and genes providing resistance to antibiotics such as vancomycin and linezolid [36,37,38] makes their use in food rather controversial.

The present work reports a search for new PEA-producing bacteria in dairy foods and assesses their potential use as starter or adjunct cultures in the manufacture of PEA-rich functional cheeses. Several strains of enterococci and lactobacilli showing the ability to produce PEA were found. Those belonging to *E. durans* were then characterized in terms of their technological properties. In this species, the production of PEA was found to be driven by the action of TDC.

## 2. Materials and Methods

### 2.1. Dairy Samples

The foods screened for PEA-producing microorganisms were four cheeses (made from different types of milk and involving different manufacturing processes) and two curd samples, all purchased at retail markets (Table 1). 

### 2.2. Bacterial Strains and Culture Conditions

Bacteria were isolated from samples of the purchased foods in GLM17 (i.e., M17 supplemented with 0.5% *w*/*v* glucose and lactose) (Formedium, Swaffham, UK), MRS (Oxoid, Basingstoke, UK), and PCA (Oxoid), all supplemented with 50 μg mL^−1^ cycloheximide (Merck, Madrid, Spain) to inhibit the growth of yeasts and moulds. All plates were incubated at 32 °C in aerobiosis for 48 h. When indicated, broths were supplemented with phenylalanine (2 mM). *E. durans* isolates were grown in GM17 (M17 supplemented with 0.5% *w*/*v* glucose) and incubated at 32 °C. *Escherichia coli* DH10B (Table 2), used for plasmid construction, was grown in LB at 37 °C with agitation. Erythromycin (5 μg mL^−1^) and/or chloramphenicol (10 μg mL^−1^) was used for *E. durans* transformant selection and plasmid maintenance, while ampicillin (100 μg mL^−1^) was used for *E. coli*.

### 2.3. Isolation of PEA-Producing Microorganisms from Dairy Samples

Screening for PEA-producing microorganisms was performed using a modified high-throughput method previously described for the selection of GABA-producing microorganisms [60]. Briefly, 1 g of cheese or curd was homogenized for 2 min in 9 mL of phosphate saline buffer (PBS) using a Lab-Blender 400 Stomacher (Seward Ltd., London, UK). Serial dilutions (10-fold) of the homogenized sample were plated on the aforementioned media (see Section 2.2) supplemented with 2% (*w*/*v*) agar. Randomly selected single colonies were picked from these serial dilutions, inoculated into 96 Deepwell plates (Thermo Fisher Scientific, Waltham, MA, USA) containing 900 μL of the corresponding medium broth, and incubated during 48 h. The plates were then centrifuged at 2500 rpm for 10 min in a 5910R Eppendorf benchtop centrifuge (Eppendorf, Hamburg, Germany) and the supernatant discarded. Cell pellets were washed three times with NaCl (0.85% *w*/*v*) and then resuspended in a reaction solution (phenylalanine 25 mM, Tween 80 0.1 mM, pH 4.7) and incubated for 24 h. After incubation, the plates were centrifuged at 2500 rpm for 10 min in the centrifuge mentioned above, and 6 μL of a pH indicator mix (50% p/v methylene blue, 50 *w*/*v* % ethyl red) were added to the supernatant. Those wells in which the colour turned green were taken, the contents diluted 10 times, and the presence of PEA was assessed by UHPLC [28].

### 2.4. DNA Manipulation

Total genomic DNA was extracted from the isolates in which PEA production was detected using the GenElute Bacterial Genomic DNA Kit (Merck), following the manufacturer’s instructions. The purified DNA was stored at −20 °C until analysis.

### 2.5. Identification of Isolates

The identification of the isolates was performed by BLAST (https://blast.ncbi.nlm.nih.gov/Blast.cgi accessed on 4 March 2025) comparison of the *16S rRNA* gene sequence obtained after PCR amplification with the primers 27F and 1492R [48] (Table 2), as previously described [61]. For *Enterococcus* isolates, species confirmation was performed by partial sequencing the superoxide dismutase gene (*sod*) with the primers sodAd1 and sodAd2 [49] (Table 2) as previously described [61].

### 2.6. RAPD-PCR Typing

Genotyping of the strains was accomplished using fingerprinting profiles obtained after RAPD-PCR with primers OLI5, 1283, and M13 (Table 2). Amplifications were performed in 25 μL reaction volumes containing 3 μL DNA (adjusted to a concentration of 60 ng/μL), 12.5 μL of 2×Master Mix RED (Ampliqon, Odense, Denmark), 5 μL of a single primer, and 4.5 μL of molecular grade water. The PCR conditions were those previously described for OLI5 [50], 1283 [51] and M13 [52], respectively. Fingerprinting amplicons were mixed with EZ-vision staining solution (Avantor, Radnor, PA, USA), separated in 2% agarose gel at 120 V for 45 min, and visualized under UV light in a V-G:Box transilluminator (Syngene, Cambridge, UK). RAPD profiles were defined based on the presence/absence of DNA bands with different electrophoretic mobilities.

Profile clustering was performed using the Unweighted Pair Group Method with Arithmetic Means algorithm (UPGMA) and Jaccard similarity coefficients, based on the RAPD-PCR profiles alone and in combination with physiological and technological traits (milk coagulating capacity, proteolytic activity, ability to utilize lactose or citrate, and production of volatile compounds and organic acids [see below]). A dendrogram was constructed using the DendroUPGMA tool [62] to reflect intraspecies differences. The final tree was generated on the iTOL webpage [63].

### 2.7. Technological Characterization

Selected *E. durans* isolates were characterized technologically via the determination of milk coagulating capacity, proteolytic activity, citrate and lactose utilization, and the production of volatile compounds and organic acids. The following assays were performed in triplicate unless otherwise stated.

#### 2.7.1. Determination of Milk Coagulating Capacity

Overnight cultures of the isolates were used to inoculate ultra-high temperature (UHT) semi-skimmed cow’s milk at 1% *v*/*v* and then incubated at 32 °C for 48 h. Milk coagulation was then assessed visually. *Lactococcus lactis* LEY6, known for its good milk coagulating capacity, was used as a positive control [40].

#### 2.7.2. Determination of Proteolytic Activity

The proteolytic activity of the isolates was examined by a qualitative method using PCA plates supplemented with 2% UHT semi-skimmed cow’s milk (Oxoid). Isolated cultures were spotted onto these plates and incubated at 32 °C for 48 h. A clear zone around the colonies indicated proteolytic activity. *L. lactis* LEY6 was again used as a positive control [40].

#### 2.7.3. Lactose and Citrate Utilization

The fermentation of lactose was assayed in BCP medium (Merck) supplemented with 1% filter-sterilized lactose. *L. lactis* LEY6, which has the ability to ferment lactose, was used as positive control [40]. Citrate utilization was assayed in a milk-based agar medium as previously reported [64]. *Lactococcus lactis* subsp. *lactis* biovar *diacetylactis* LA1, which has a citrate–utilizing phenotype, was used as a positive control [52].

#### 2.7.4. Production of Volatile Compounds

Volatile compounds in the fermented milks were quantified by headspace/gas chromatography/mass spectrometry (HS/GC/MS) using an Agilent 8890 apparatus (Agilent Technologies, Wilmington, DE, USA) equipped with an MSD 5977B detector (Agilent Technologies) and an HP-Innowax column (30 m × 250 μm × 0.25 μm; Agilent Technologies). Fermented milks were prepared by inoculating 20 mL SPME chromatographic vials (with a magnetic septum and PTFE/silicone screw cap) (Agilent Technologies) containing 5 mL of UHT semi-skimmed milk and a 1% inoculum of an overnight culture of each strain (incubated at 32 °C for 72 h and then frozen at −80 °C until use). These vials were equilibrated for 10 min at 60 °C under pulsed agitation (5 s at 500 rpm) in a PAL RSI 120 (CTC Analytics AG, Zwingen, Switzerland). Volatile compounds were absorbed onto a DVB/PDMS ARR11-DVB-120/20 fibre (CTC Technologies, Ann Arbor, MI, USA) introduced into the head space vial over the samples (40 mm deep, 60 °C, 20 min). The fibre was then injected into a GC spectrophotometer with a split/splitless injector in splitless mode, and deorbited for 1 min at 250 °C. The column oven temperature was adjusted to 35 °C maintained for 30 s, with an increment of 6.5 °C min^−1^ until reaching 165 °C; thereafter the increment was 20 °C min^−1^ until reaching 260 °C. The transit time was 26.5 min. Helium, at a flux of 1.4 mL min^−1^, was used as the mobile gas phase. The mass detector was used in simple quadrupole mode; the ion source temperature was set to 230 °C while that of the interface was set to 280 °C. The EM mode was set as electronic ionization (70 eV), with a mass scanning range of 35 to 250 amu. GCMS auto-tuning (ETUNE-F1) was performed prior to analysis to ensure optimal GCMS performance. Compounds were identified by comparing their mass spectra with those in the NIST 2014 database. Identified peaks with a similarity score of over 600 were quantified as the normalized value of the chromatogram peak area with respect to the total area of the sample. Negative controls (non-inoculated UHT semi-skimmed cow’s milk samples) were performed in parallel. 

#### 2.7.5. Production of Organic Acids

UHT semi-skimmed cow’s milk was inoculated in triplicate at 1% with overnight cultures of each strain and incubated for 48 h. The organic acids produced during growth were determined by HPLC following a previously described method [52]. Briefly, compounds were separated in an ICSep ICE-ION-300 ion-exchange column (Waters, Waltham, MA, USA), with 8.5 mN H_2_SO_4_ as the mobile phase (operating temperature 65 °C, flow rate 0.4 mL min^−1^). Sugars were identified using a Waters model 410 differential refractometer at 280 nm, and organic acids using a Waters model 996 photodiode array detector at 210 nm (Waters). The concentration of individual metabolites was obtained using calibration curves prepared with commercial standards.

### 2.8. Safety Evaluation

#### 2.8.1. Antimicrobial Resistance

Antimicrobial susceptibility was examined via the disc diffusion method. Overnight cultures were adjusted to a 0.5 McFarland turbidity standard and 100 μL of the cell suspension spread onto Muller–Hinton agar plates (Oxoid). Nine antibiotic discs—chloramphenicol (30 μg/disc), erythromycin (15 μg/disc), gentamicin (10 μg/disc), kanamycin (30 μg/disc), streptomycin (10 μg/disc), vancomycin (30 μg/disc), ampicillin (10 μg/disc), clindamycin (2 μg/disc), and tetracycline (30 μg/disc) (all from Oxoid)—were deposited on the plates which were then incubated at 37 °C for 20–24 h. The diameter of any clear zone was then measured, and the susceptibility of the isolates interpreted according to the criteria of the European Committee on Antimicrobial Susceptibility Testing Standard v_14.0, and the European Food Safety Authority (EFSA) [65]. *E. faecalis* CECT 795 was used as an indicator strain for antibiotic susceptibility.

Additionally, the minimum inhibitory concentration (MIC) of ampicillin was assessed (as recommended by EFSA for *Enterococcus* spp.) using the broth microdilution method in 96-well microtitre plates containing Mueller–Hinton broth [66]. The range of ampicillin concentrations tested varied between 0.125 and 64 μg mL^−1^ using a dilution factor of two for each well. Plates were inoculated to attain a final cell concentration of 5.5 × 10^5^ cfu/mL and then incubated at 37 °C for 20 h. The MIC values were determined as the first well in which no growth was observed.

#### 2.8.2. Presence of Virulence-Related Genes

The presence of the virulence factors *gelE* (extracellular metalloendopeptidase, which hydrolyses gelatine, collagen and hemoglobin), *efaA* (a cell wall adhesin expressed in serum), *esp* (a cell wall–associated protein involved in immune evasion), *hylEfam* (a glycosyl hydrolase involved in virulence) and *IS16* (an insertion element associated with virulent hospital strains) was examined by specific PCR (Table 2) as recommended by EFSA [66], using the primers and conditions previously described [57,58,59]. PCR products were separated in 0.8% agarose gels and visualized as described above. *E. faecium* VR1 was used as a positive control for *efaA*, *esp* and *IS16*, *E. faecalis* CECT 795 for *gelE*, and *E. faecium* DAPTO R for *hylEfam* (Table 2).

#### 2.8.3. Production of Biogenic Amines

The capacity to produce the BAs tyramine, histamine and putrescine was assessed in broth, and the presence of the genes involved in their production (*tdcA* for tyramine, *hdcA* for histamine, and *odc* or *agdI* for putrescine) examined by PCR.

Biogenic amine production in broth was measured by UHPLC as previously described [28] in supernatants obtained after the incubation of *E. durans* isolates for 48 h in 10 mL of GM17 broth supplemented with either 1 mM tyrosine, 1 mM histidine, 1 mM ornithine or 1 mM agmatine (ornithine and agmatine are precursors of putrescine via different pathways), as previously described [61].

The presence of the genes involved in BA production was examined by PCR amplification of total DNA from the isolates using specific primers (Table 2). The PCR conditions were those previously described for *tdcA* [53], *hdcA* [54], *odc* [56] and *AgdI* [55]. Positive controls were set up using total genomic DNA obtained from different BA-producing strains: *E. faecalis* V583 for *tdc* and *AgdI* [42], *Lentilactobacillus parabuchneri* IPLA11150 for *hdcA* [43] and *Furfurilactobacillus rossiae* D87 for *odc* [44]. PCR products were separated in 0.8% agarose gels and visualized as described above.

### 2.9. Construction of a tdcA Mutant

An *E. durans* IPLA655 derivative mutant strain deficient in the *tdcA* gene (i.e., with an internal deletion in *tdcA* from nt 52 from the start codon to nt 1831) but replaced by the chloramphenicol resistance gene (*cat*) from pMN1 vector [47] was constructed by double crossover homologous recombination following a previously described protocol [47]. Briefly, the flanking fragments of the *tdcA* gene were amplified by PCR using primers KOtdcUpEcoRI and KOtdcUpBamHI for the upstream fragment, and KOtdcDwBAmHI and KOtdcDwHindIII for the downstream fragment (Table 2). The amplicons were purified and subsequently cloned in *E. coli* DH10B cells, flanking the *cat* gene in EcoRI-BamHI and the BglII-HindIII sites of pMN1, respectively, to generate pIPLA1305. pIPLA1305 was transformed into electrocompetent *E. durans* IPLA655 cells obtained as previously described [67]. Transformants were selected on GM17 plates supplemented with erythromycin and chloramphenicol. *E. durans* IPLA655 harbouring pIPLA1305 in the chromosome was grown in GM17 supplemented with chloramphenicol to select bacteria, showing evidence of double-crossover events. The replacement of the *tdcA* gene by the *cat* gene was checked by PCR amplification and further sequencing (Macrogen, Madrid, Spain), making use of the primers 655DtdcUP and 655DtdcDW (Table 2). The absence of tyramine and PEA biosynthesis was checked by UHPLC [28] analysis of the supernatants of overnight cultures in GM17 supplemented with tyrosine (2 mM) or phenylalanine (2 mM), as described above.

## 3. Results

### 3.1. Isolation of PEA-Producing Strains

A total of 1316 bacterial isolates from the different dairy samples and grown in the different media (564 in MRS, 564 in GLM17, and 188 in PCA) were tested for their ability to produce PEA (Table 1). Sixty-three isolates (4.8%) were selected based on the colour change (both strong and weak) in the well after the addition of the pH indicator mix. Those wells in which the colour change was not as evident were anyhow included to maximize the number of potential PEA-producers. The largest number of positive wells were obtained from the Terrincho and Cabrales cheese samples, followed by that of Roncal. Positive results were also obtained with the cow’s milk curd. No positive wells were obtained from the very long-ripened sheep’s cheese (Table 1). Selected wells were analyzed for the presence of PEA in the supernatant by UHPLC. Forty-one (65%) of the analyzed wells showed PEA production had occurred (Table 3). 

### 3.2. Verification of PEA-Producing Capacity in Culture Broth

Isolates present in PEA-containing wells were grown in the corresponding isolation broth (supplemented with 2 mM phenylalanine) to check their capacity to produce PEA. After 48 h of incubation, the supernatants were examined for the presence of PEA by UHPLC. Thirty-three of the 41 isolates tested produced PEA in broth and were selected for further work. Although most (33 [80%]) of the isolates tested showed the capacity to produce PEA in broth, some (8 out of 41) were unable to produce PEA under the culture conditions tested. 

### 3.3. PEA-Producing Isolates: Identification at the Species Level by 16S rRNA Gene Sequencing

Total DNA from the 33 PEA-producing isolates was obtained and used for species identification via *16S rRNA* gene sequence analysis and BLAST comparison. For the *Enterococcus* isolates, the species assignment was confirmed by *sodA1* gene sequence comparison (identification at the species level is not always accurate for this genus using *16S rRNA* analysis alone) [49,61]. The 33 isolates were identified as belonging to *E. durans* (13 isolates), *E. faecalis* (8 isolates), *E. faecium* (8 isolates), and *L. brevis* (4 isolates) (Table 3). *E. faecalis* and *E. faecium* are controversial in terms of their biotechnological use, especially those related to food, given their pathogenic potential and their role in spreading antimicrobial resistance genes [68]. They were thus excluded from further analysis. The amount of PEA detected in the supernatants of the *L. brevis* isolates was much lower than for the *E. durans* isolates (Table 4), so they were also excluded from further examination. 

Based on these considerations, the larger number of PEA-producing *E. durans* isolates, and their greater production capacity, the 13 *E. durans* isolates were selected for further characterization. The *E. durans* IPLA655 strain, previously characterized by our group, and for which the genome is available [46], was included in the study for comparative purposes after testing its ability to produce PEA.

### 3.4. RAPD-PCR Typing

**Figure 1:**
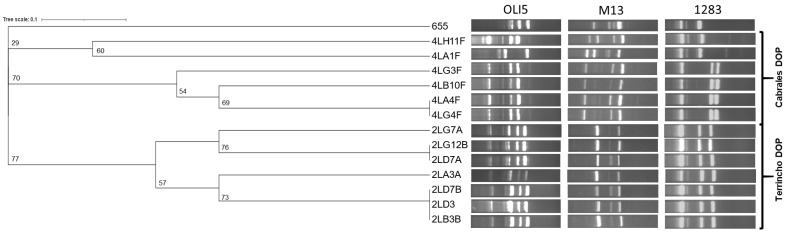
Similarity dendrogram based on the combined RAPD typing profiles of the PEA-producing *Enterococcus durans* isolates obtained with primers OLI5, M13 and 1283. Clustering was performed via the unweighted pair group method using arithmetic averages (UPGMA) and Jaccard similarity coefficients. The source of the isolates is indicated with brackets. Bootstrap values, based on 100 replicates, are indicated at the corresponding branch nodes.

RAPD was used to determine the genetic biodiversity of all the *E. durans* isolates, including *E. durans* IPLA655. Ten candidate strains were detected. Isolates with the same origin tended to cluster closely (Figure 1).

### 3.5. Technological Characterization of PEA-Producing E. durans Isolates

Given the large phenotypic variation reported for closely related strains of *Enterococcus* [69], and the manageable number of PEA-producing *E. durans* isolates, all of them were subjected to technological characterization.

#### 3.5.1. Lactose and Citrate Utilization

All the isolates showed the capacity to ferment lactose in the BCP broth test, as evidenced by the colour change (Table 5). In addition, the consumption of lactose in milk was also examined by HPLC. All of the isolates were able to partially consume lactose (10–25%), except for 4LB10F, 4LG4F, 2LD3 and 2LG7A (no consumption observed at all) (Table 6). At 65%, the partial consumption of lactose by isolate 4LA1F is notable. Most of the isolates were able to consume free residual glucose or galactose in the milk sample (Table 6).

None of the isolates consumed citrate in the plate test, as indicated by the absence of the colour change that appeared in the positive control with *L. lactis* subsp. *lactis* biovar *diacetylactis* LA1 (Table 5). 

#### 3.5.2. Proteolytic Activity

All of the *E. durans* isolates showed the capacity to hydrolyse casein, as evidenced by the appearance of a clear halo in the milk plate test, with the exception of isolates 2LA3A and 2LG7A (Table 5; Supplementary Figure S1). The largest halo diameters were obtained with isolates 4LA1F, 4LA4F, 4LB10F, 4LG3F, 4LG4F, 2LB3B, 2LD3, and 2LG12B; the smallest were observed for 4LH11F, 2LD7A, 2LD7B, and IPLA655 (Table 5).

#### 3.5.3. Milk Clotting Capacity

Of the 14 isolates tested, only 4LA1F and 4LH1F were able to cause some coagulation of milk after 48 h of incubation at 32 °C. Compared to the coagulation produced by the control strain, the one caused by 4LA1F was moderate and the one by 4LH11F was small (Table 5).

#### 3.5.4. Production of Volatile Compounds

**Figure 2:**
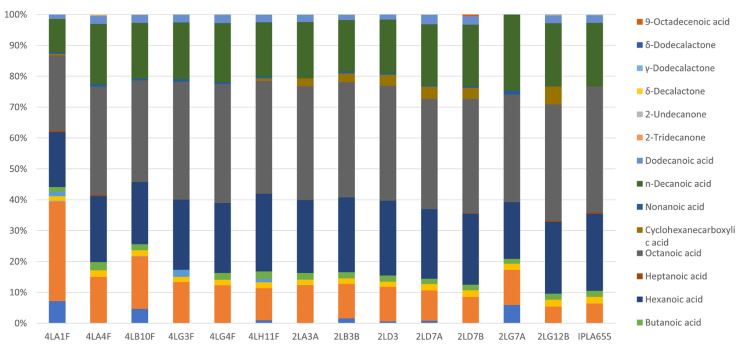
Relative abundance of different volatile compounds produced in milk by PEA-producing *Enterococcus durans* isolates.

The concentrations of the different volatile compounds identified in the milk before and after inoculation with the different strains of *E. durans* are shown in Table 7. The 26 detected compounds were grouped according to the following classes: aldehydes (2), ketones (6), lactones (3), acids (12), alcohols (2), and sulphur compounds (1). While ketones and acids were the main constituents of non-inoculated milk (relative abundances 45% and 40%, respectively), in the fermented milk, this profile altered drastically, with the acid group becoming the majority (90%) and with ketones reduced to 7%. Some compounds tended to be consumed (acetaldehyde, benzaldehyde, 2-pentadecanone, and 2-heptanone) while others were produced, mainly acetic acid, butanoic acid, hexanoic acid, octanoic acid, nonanoic acid, n-decanoic acid, 9-decenoic acid, dodecanoic acid, and acetoin. Figure 2 shows the relative abundance of the compounds produced by each of the *E. durans* strains. Overall, no drastic differences were observed among the isolates, with the main compounds produced being acetic acid (ranging from 5.4% to 30.4%), octanoic acid (23.0–37.9%) and hexanoic acid (16.8–25.2%). However, some compounds were not produced by all strains. One of the most important was acetoin, a compound of great interest, since it is associated with a pleasant buttery aroma; this was only produced by strains 4LA1F, 2LB3B, 2LD3, 2LD7A, and 2LG7A (0.9–6.7%). Benzoic acid, which was produced by strains 4LA1F, 4LG3F, and 4LH11F (1.2–1.3%), is associated with pleasant aromas. Cyclohexanecarboxylic acid, which is associated with fruity aromas, was produced only by 4LA1F, 4LH11F, 2LA3A, 2LB3B, 2LD3, 2LD7A, 2LD7B, and 2LG12B (0.5–5.8%).

#### 3.5.5. Production of Organic Acids

Small amounts of pyruvic acid (1–5 mg/mL), acetic acid (19–90 mg/mL), and lactic acid (155–405 mg/mL) were produced by all isolates. All were able to produce citrate (276–412 mg/mL), except for 4LA1F. The production of organic acids is related to the consumption of sugars present in the milk, mainly lactose, which was partially consumed by most of the isolates (see Section 3.5.1). Clearly, the diversity and quantity of organic acids produced depends on each isolate’s metabolic capacities.

### 3.6. Safety Assessment of PEA-Producing E. durans Isolates

#### 3.6.1. Antibiotic Susceptibility

All the isolates were sensitive to ampicillin, gentamicin, kanamycin, chloramphenicol, tetracycline, and vancomycin. Variable susceptibility to streptomycin, erythromycin, and clindamycin was noted. Isolate 4LA4F was clindamycin-resistant, 4LB10F, 2LG12B, and IPLA655 were streptomycin- and clindamycin-resistant, and 4LG4F was erythromycin- and clindamycin-resistant. Isolate 4LG3F was resistant to streptomycin, erythromycin, and clindamycin (Table 8).

EFSA guidelines indicate that *Enterococcus* species must be susceptible to ampicillin (MIC ≤ 2 mg/L) if they are to be used in food applications. All the isolates were sensitive and had associated MIC values under EFSA cut-off point, as determined using the microbroth dilution method (Table 9).

#### 3.6.2. Virulence Factors

Following EFSA guidelines for assessing the safety of *Enterococcus*, all the isolates were screened for the presence of *IS16*, *esp*, and *hylEfam* virulence factors, as well as *gelE* and *efaA*. None of these was detected in any isolate, with the exception of *esp* in 2LD7A (Table 10).

#### 3.6.3. Production of Biogenic Amines

All the isolates were tested for their ability to produce the BAs histamine, tyramine and putrescine, and for the corresponding genes (*hdcA*, *tdcA*, and *odc* or *agdI,* respectively). None of the isolates returned a positive PCR test for the presence of the genes related to the production of histamine or putrescine (for the latter, neither of the two alternative production pathways [ODC or AGDI] was represented) [44]. Neither histamine nor putrescine was detected in media supplemented with the precursor aminoacids histidine, ornithine or agmatine. However, all the isolates were positive for the presence of the *tdcA* gene involved in the production of tyramine, and, consequently, tyramine was detected in the culture supernatants of all the isolates grown in GM17 broth supplemented with tyrosine (Table 11). 

### 3.7. Typing of PEA-Producing E. durans Isolates

**Figure 3:**
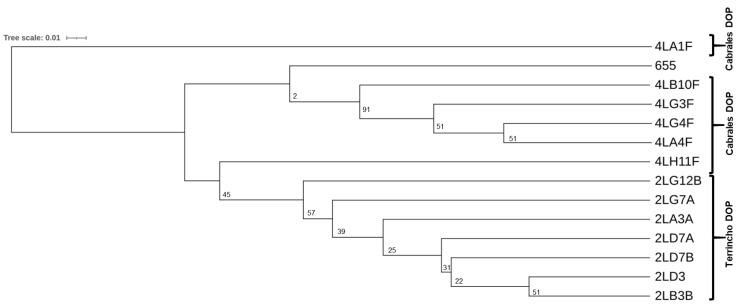
Similarity dendrogram based on the RAPD-PCR profiles in combination with the technological traits of the PEA-producing *Enterococcus durans* isolates. Clustering was performed via the unweighted pair group method using arithmetic averages (UPGMA) and Jaccard similarity coefficients. The source of the isolates is indicated with brackets. Bootstrap values, based on 100 replicates, are indicated at the corresponding branch nodes.

For more precise typing of the *E. durans* isolates, clustering was performed using the UPGMA (Figure 3); this involved data obtained in the RAPD-PCR and technological and safety analyses (i.e., milk coagulating capacity, proteolytic activity, ability to utilize lactose or citrate, production of volatile compounds, and safety profile). According to this analysis, all of the isolates corresponded to different strains and tended to aggregate according to their origin.

### 3.8. The Tyrosine Decarboxylase Gene (tdcA) Is Responsible for PEA Production in E. durans

The literature contains no mention of any specific phenylalanine decarboxylase involved in PEA production; indeed, for *E. faecium*, the conversion of phenylalanine into PEA has only been recorded as occurring via tyrosine decarboxylase, which is encoded by the gene *tdcA* [33]. To check whether this gene is responsible for the production of PEA in *E. durans*, it was knocked out in *E. durans* IPLA655, a strain for which a genome sequence is available [46], and for which a transformation protocol has been optimized in our laboratory [67]. Cloning of the *tdcA* gene flanking regions in the suicide vector pMN1 allowed for the replacement of *tdcA* gene by *cat*, resulting in the strain *E. durans* IPLA655*ΔtdcA* (Table 12). The genotype of the new mutant strains was confirmed by PCR and sequencing of the amplified region. Its phenotype was assayed in vivo after UHPLC analysis of the supernatants in GM17 broth supplemented with tyrosine (2 mM) or phenylalanine (2 mM). The *E. durans* IPLA655 *ΔtdcA* strain produced neither tyramine nor PEA in broth, confirming the implication of the tyrosine decarboxylase (and thus *tdcA*) in the production of PEA from phenylalanine. 

## 4. Discussion

PEA is a neuroactive trace amine involved in the regulation of mood and attention [5,70] and is marketed as a food supplement for improving mood and athletic performance [14]. In this context, the development of PEA-enriched functional foods is of some interest that aligns with the growing consumer interest in functional foods that support mental well-being [11]. In this regard, cheese offers an exceptional matrix for its accumulation if PEA-producing microorganisms are present [25]. The integration of PEA producing microorganisms in dairy products offers a novel strategy to develop tailored functional foods with neuromodulatory potential. The in situ production of PEA circumvents challenges associated with direct PEA fortification, as integration in the food matrix, while increasing consumer acceptance that look for more natural and healthy products [71]. However, the selection of microorganisms is crucial to ensure efficient and secure PEA yield, compatibility with the dairy elaboration process, and desirable final organoleptic properties while maintaining food safety.

In the present work, 33 isolates that produced PEA in broth supplemented with phenylalanine were isolated from dairy samples. Although 63 isolates were initially selected in the screening method, the presence of PEA was not detected in all of them. It should be remembered that the selection of wells to be analyzed included those in which the colour change in the pH indicator was weak, which would explain the absence of PEA in some wells. Production of BA are favoured by certain environmental conditions such as pH, carbon source, temperature, among others [72,73,74]. In our test, we did not optimize growth conditions or other parameters, except for the addition of the precursor amino acid (phenylalanine), which could explain why some isolates were shown to produce PEA in the screening conditions (resting cells at acidic pH with phenylalanine) but not in broth.

PEA-producing strains of *L. brevis* (4), *E. faecalis* (8), *E. faecium* (8), and *E. durans* (13). were isolated. To our knowledge, this is the first time that PEA-producing strains of *L. brevis* of dairy origin have been described as PEA producers. The capacity to produce PEA has been previously described in *L. brevis* strains isolated from wine [32]. However, they showed a limited capacity to produce PEA in the conditions tested and were then discarded for further analysis. While *E. faecalis* and *E. faecium* have been reported to synthesize this compound [31], as have *Enterococcus mundtii* [75] and *Enterococcus hirae* [34], the literature contains scant information on *E. durans* as a PEA-producer.

*E. durans* is a natural member of the cheese microbiota that can reach elevated numbers, especially in artisanal mediterranean cheeses [76]. *E. durans*, as well as other enterococcal species, plays a role in the development of cheese flavour and texture through its proteolytic and esterolytic activities due to the production of short- and medium-chain fatty acids, acetaldehyde, acetoin, and diacetyl compounds [76,77]. In addition, they can also be considered as a protective adjunct cultures thanks to the capacity of some strains to produce enterocins (broad-spectrum bacteriocins) with antimicrobial activity against pathogenic and spoilage microorganisms [78]. Taking into account these beneficial properties, we have selected this species for technological characterization to determine their potential as starter or adjunct cultures for the production of PEA-rich functional cheeses based on their higher PEA production (Table 4) and the reported positive effects in foods including cheese [79,80]. In recent years, this species has been of increasing interest from a biotechnological point of view. Although belonging to the *E. faecium* group, it carries almost no associated virulence or AMR genes [81], has a number of interesting probiotic and technological properties [82], and is certainly prevalent in dairy products [83], including a number of different cheese types [80,84]. 

Examination of the present isolates’ genetic and technological traits revealed wide diversity among them; indeed, they were all different strains (one of which was the previously characterized *E. durans* IPLA 655). Certainly, their provenance was a source of this variation; strains isolated from the same cheese were more similar to one another than were strains isolates from different cheeses (Figure 3). The technological behaviour of the strains was quite similar, reflecting their adaptation to the dairy environment. For example, most were able to ferment lactose, both in the BCP test (Table 5) and, most importantly, when grown in milk (Table 6). Some strains could not utilize the lactose in milk, but in BCP broth they were able to ferment it. Milk is a more complex medium in which not all microorganisms can grow. 

Twelve of the fourteen strains studied for proteolytic activity (which has a major impact on cheese texture and flavour) returned positive results, although the intensity varied depending on the strain (Table 5; Supplementary Figure S1). Only two strains caused milk to coagulate—a consequence of acidification and caseinolytic capacity—albeit incompletely. Since proteolytic and milk coagulating capacity are essential properties of starter cultures, the present strains might be of limited use in this respect.

None of the strains showed the ability to utilize citrate under the present test conditions. Indeed, few strains of *E. durans* have ever been reported to consume citrate or to carry the necessary genes (*cit* operon) to be able to do so [79,85]. This is somewhat negative from a cheese technology point of view since its utilization is related to the formation of key aroma compounds, such as diacetyl and acetoin. However, all the strains showed the ability to produce citrate when grown in milk (Table 6). This citrate could be used by other bacteria present in the cheese matrix, as a cross-feeding strategy, allowing them to produce diacetyl, acetoin, or other aromatic compounds [86]. In spite of their inability to use citrate, all the PEA-producing strains analyzed produced volatile aromatic compounds and acids in milk, positive aspects from the standpoint of their potential use as adjunct cultures. The aromatic compounds produced included hexanoic acid, octanoic acid, n-decanoic acid, acetic acid, citric acid, lactic acid, and, of particular interest, acetoin. Given the above properties the use of the analyzed strains as starter cultures does not seem appropriate. However, they may be of great interest as adjunct cultures to develop interesting flavour characteristics during ripening [77].

Concerning safety analysis, in microorganisms for food application, it is particularly interesting to investigate the presence of virulence related genes as well as the antibiotic resistance profile. In recent years, the isolation of antibiotic-resistant *Enterococci* from food products and food environments has increased globally [87]. However, different studies have shown that antibiotic resistance and the presence of virulence factors are strain-specific in food isolates and in the case of cheeses, there are significant variations between countries, cheese varieties, and from raw material to final product. [36,80,88]. Most enterococci of dairy origin have been reported susceptible to ampicillin, chloramphenicol, gentamicin, penicillin, and tetracycline [78,88,89,90], and to show some degree of resistance to erythromycin and ciprofloxacin [36,91]. The present strains returned similar results. Although the resistance profiles did not vary drastically (all were sensitive to ampicillin, gentamicin, chloramphenicol, kanamycin, vancomycin, and tetracycline), some were resistant to streptomycin, erythromycin and/or clindamycin. This would preclude their use as food additives unless genome analyses can show these resistances not to be associated with elements that mobilize, such as plasmids or integrons. That said, eight strains showed no resistance to any of the antibiotics tested and could potentially be safely used. 

Since enterococci are commonly considered opportunistic pathogens, their carriage of the most important virulence genes was examined. All strains were negative for the *gelE*, *efaA*, *hylEfam* and *IS16* virulence factors, and only one carried the *esp* gene. The latter, which has been implicated in biofilm formation [92], usually lies within a large pathogenicity island [93,94]. According to EFSA guidelines, *esp* is a particularly negative marker in terms of food safety, precluding any strain that carries it from use in food applications. Notwithstanding this concern, the involvement of this gene in adhesion and biofilm formation might render its carriers of interest as gut colonizing probiotics [95].

Although none of the strains produced the BAs histamine or putrescine, all of them produced tyramine. The ability of *E. durans* to produce tyramine has been reported by other authors [96,97,98]. In fact, *E. durans* IPLA655, a strain studied in the present work, has been extensively examined as a model for tyramine production and its genetic regulation [67]. Indeed, tyramine production is a species-level trait of *E. durans*, as it is in *E. faecalis* and *E. faecium* [99].

PEA production has been previously described in other species as the result of the decarboxylation of phenylalanine through the action of TDC [31]. This activity has only been described and characterized in Gram-positive bacteria—more specifically in lactic acid bacteria (LAB) within the genera *Enterococcus*, *Lactobacillus*, *Leuconostoc*, *Lactococcus,* and *Carnobacterium* [31] (Supplementary Figure S2). All of these can be present during the manufacture of fermented food products. However, the capacity of TDC to decarboxylate phenylalanine—an amino acid structurally related to tyrosine—seems to depend not only on species or strain but on environmental conditions [31]. As previously shown for *E. faecium* [33], tyrosine/phenylalanine decarboxylase activity (via TDC, encoded by *tdc*) was detected in *E. durans* via the generation of the mutant *E. durans* IPLA655 *Δtdc*, which lost the ability to produce tyramine and PEA.

The good technological properties shown by the present PEA-producing *E. durans* strains, plus the absence of antibiotic resistance and virulence genes from most of them, suggests that they may be safe for use in food applications, and particularly in the production of PEA-rich functional cheeses. However, their production of tyramine is an obstacle. In humans, the ingestion of excess tyramine can lead to vasoconstriction, hypertension, nausea, vomiting and headaches—a phenomenon known as the ‘cheese reaction’ [25,100]. Moreover, tyramine has been described as cytotoxic in concentrations that can be found in certain foods, mainly cheeses [101]. Although there is no legal limitation on the presence of tyramine or other BA in dairy products, there is a recommendation to reduce their concentration in foods to avoid the ingestion of ≥600 mg of tyramine per meal, a threshold concentration that, if overpassed, may result in intoxication symptoms in healthy individuals [24]. To overcome this problem, the optimal conditions for PEA production and reduced production of tyramine needs first to be determined since the pH, temperature, and NaCl concentration can significantly affect PEA and toxic tyramine production. For example, PEA is the main amine produced by *E. faecalis EF37* in skimmed milk, whereas in broth it produces tyramine in larger quantities [102]. Further, in fermented sausages, the same strain has been shown to accumulate PEA over ripening, when tyrosine becomes a limiting substrate [103]. This opens up the possibility of modifying different variables during the cheese making process to minimize tyramine accumulation and promote PEA accumulation. 

The technological and safety characterization of the *E. durans* strains showed that they might be candidates to use as cheese adjunct cultures for the elaboration of PEA-rich functional dairy products. However, several challenges have risen for the development of such products. Although PEA has the GRAS status by FDA [104] and it has been approved as food flavouring agent at EU [105], ingestion of elevated concentrations of PEA can provoke toxicological symptoms [24]. Therefore, the technological conditions to obtain dairy products with adequate final concentrations of PEA should be determined. PEA, as well as other BA, is stable in the food matrix and can resist also heating processes [106], which would be an advantage to maintain constant levels once produced. The production of tyramine—a toxic BA—due to the fact that TDC is involved in the production of PEA and tyramine in *E. durans*, is another challenging issue that must be investigated to determine the optimal fermentation conditions to obtain low concentrations of tyramine as well as an adequate concentration of PEA. Finally, although there is an increasing demand and acceptance of functional foods by consumers that look for more natural and healthy products [71], consumer acceptance should also be studied.

## 5. Conclusions

Thirty-three PEA-producing isolates from different dairy products were identified as belonging to *L. brevis*, *E. durans*, *E. faecium,* and *E. faecalis*. The technological characterization of the *E. durans* strains, which showed greater capacity to produce PEA, revealed that they might be candidates for use as cheese adjunct cultures. The production of PEA by these strains was shown to involve TDC, encoded by *tdcA*, by the generation of a *ΔtdcA* knockout mutant.

## Figures and Tables

**Table 1:** Characteristics of the dairy samples screened for PEA-producing microorganisms.

Sample	Milk Type	Milk Treatment	Ripening Period	Number of Colonies Picked Off			Positive Colour Change
				MRS	LGM17	PCA	
Terrincho DOP	Sheep	Raw	Medium	94	94	-	23
Roncal DOP	Sheep	Raw	Long	94	94	-	9
Cabrales DOP	Cow, Sheep, Goat	Raw	Long	94	94	-	19
Sheep Cheese	Sheep	Raw	Very long	94	94	-	0
Cow’s milk curd	Cow	Raw	Very short	94	94	94	12
Sheep’s milk curd	Sheep	Raw	Very short	94	94	94	0

DOP, Protected Designation of Origin.

**Table 2:** Bacterial strains, constructs, plasmids, and primers.

Strains	Relevant Genotype, Description, or Properties	Reference or Source
*E. coli* DH10B	F–, *mcrA* Δ(*mrr-hsdRMS-mcrBC*), ϕ80*lacZ*ΔM15, Δ*lacX74*, *recA1*, *endA1*, *araD139*, Δ (*ara-leu*)7697, *galU*, *galK*, λ–*rpsL*(Str^R^), *nupG*	[39]
*E. coli* IPLA1305	*E. coli* DH10B harbouring pIPLA1305; Amp^R^, Ery^R^, Cm^R^	This work
*Lactococcus lactis* LEY6	Positive control for technological traits	[40]
*L. lactis* subsp. *lactis biovar diacetylactis* LA1	Citrate utilization positive control	[41]
*E. faecalis* V583	Tyramine- and putrescine-producing strain	[42]
*L. parabuchneri* IPLA11150	Histamine-producing strain	[43]
*F. rossiae* D87	Putrescine-producing strain	[44]
*E. faecalis* CECT 795	Strain carrying *gelE* gene, indicator of antibiotic susceptibility	CECT
*E. faecium* VR1	Strain carrying *efaA*, *esp* and *IS 16* genes	[45]
*E. faecium* DAPTO R	Strain carrying *hylEfam*	[45]
*E. durans* IPLA655	Tyramine- and PEA-producing strain	[46]
*E. durans* IPLA655-*Δtdc*	*E. durans* 655 derivative in which the *tdc* gene was replaced with the *cat* gene; Cm^R^	This work
**Plasmids**	**Relevant genotype, description or properties**	**Reference or source**
pMN1	Cloning vector designed to make knockout mutants in Gram-positive bacteria; Amp^R^, Ery^R^, Cm^R^	[47]
pIPLA1305	pMN1 with upstream and downstream flanking regions of *tdc* gene; Amp^R^, Ery^R^, Cm^R^	This work
**Primers**	**Sequence 5′-3′**	**Function/reference**
27F	AGAGTTTGATCMTGGCTCAG	Species identification [48]
1492R	TACGGYTACCTTGTTACGACTT	Species identification [48]
*sodA1*	CCITAYICITAYGAYGCIYTIGARCC	Identification of enterococcal species [49]
*sodA2*	ARRTARTAIGCRTGYTCCCAIACRTC	Identification of enterococcal species [49]
OLI5	AACGCGCAAC	Typification of the isolates by RAPD [50]
1283	GCGATCCCCA	Typification of the isolates by RAPD [51]
M13	GAGGGTGGCGGTTCT	Typing of the isolates by RAPD [52]
tdc1	AACTATCGTATGGATATCAACG	Detection of the *tdcA* gene [53]
tdc2	TAGTCAACCATATTGAAATCTGG	Detection of the *tdcA* gene [53]
hdcDG-F	CCTGGTCAAGGCTATGGTGTATGGTC	Detection of the *hdcA* gene [54]
hdcDG-R	GGTTTCATCATTGCGTGTGCAAA	Detection of the *hdcA* gene [54]
AgmSq1	CAAGATTTDTTCTGGGCHTTYTTCTC	Detection of the *AgdI* cluster [55]
AgmSq1	TTGGHCCACARTCACGAACCCT	Detection of the *AgdI* cluster [55]
ODC1	NCAYAARCAACAAGYNGG	Detection of the *odc* gene [56]
ODC2	GRTANGGNTNNGCACCTTC	Detection of the *odc* gene [56]
TE9	ACCCCGTATCATTGGTTT	Detection of the *gelE* gene [57]
TE10	ACGCATTGCTTTTCCATC	Detection of the *gelE* gene [57]
TE34	TTGCTAATGCTAGTCCACGACC	Detection of the *esp* gene [57]
TE36	GCGTCAACACTTGCATTGCCGAA	Detection of the *esp* gene [57]
TE37	AACAGATCCGCATGAATA	Detection of the *efa* gene [57]
TE38	CATTTCATCATCTGATAGTA	Detection of the *efa* gene [57]
IS16-F	CATGTTCCACGAACCAGAG	Detection of *IS16* gene [58]
IS16-R	TCAAAAAGTGGGCTTGGC	Detection of the *IS16* gene [58]
hylEfm-F	GAGTAGAGGAATATCTTAGC	Detection of the *hylEfm* gene [59]
hylEfm-R	AGGCTCCAATTCTGT	Detection of the *hylEfm* gene [59]
KOtdcUpEcoRI,	CCCCATTTTGAATTCTACCAATTC	Amplification of the upstream *tdc* flanking regions (this work)
KOtdcUpBamHI,	GGGGGATCCGTTCTCAGCTTTGTCCCCG	Amplification of the upstream *tdc* flanking regions (this work)
KOtdcDwBamHI	GGGGGATCCAAATCTACGCAGATCAATTATTAGC	Amplification of the downstream *tdc* flanking regions (this work)
KOtdcDwHindIII	CTGGAAAAGCTTTTGACCAAGAGAAGTCACC	Amplification of the downstream *tdc* flanking regions (this work)
655DtdcUP	GGTTGTCGTTAATACAATCC	Verification of the *E. durans* 655 *Δtdc* genotype (this work)
655DtdcDW	CAATAACCGAAAGCAAACAG	Verification of the *E. durans* 655 *Δtdc* genotype (this work)

AmpR, ampicillin resistance; EryR, erythromycin resistance; CmR, Chloramphenicol resistance; CECT, Spanish Type Culture Collection. Restriction sites are underlined.

**Table 3:** PEA-producing isolates identified in the positive dairy samples.

Sample	Analyzed Wells	PEA Producers in Resting Cells (Well)	PEA Producers in Broth	Species Identification (Number)
Terrincho	23	23	15	*L. brevis* (2)
				*E. faecalis* (2)
				*E. faecium* (4)
				*E. durans* (7)
Roncal	9	2	2	*E. faecalis* (2)
Cabrales	19	11	11	*L. brevis* (2)
				*E. faecalis* (1)
				*E. faecium* (2)
				*E. durans* (6)
Cow’s milk curd	12	5	5	*E. faecalis* (3)
				*E. faecium* (2)

**Table 4:** β-phenylethylamine (PEA) production by identified isolates grown in isolation broth supplemented with 2 mM phenylalanine for 48 h.

Species	Origin	Isolate	PEA (mM)
*Enterococcus faecalis*	Terrincho DOP	2LG2	1.0 ± 0.1
*Enterococcus faecalis*	Terrincho DOP	2LH4	1.2 ± 0.2
*Enterococcus faecalis*	Roncal DOP	3LB10	0.5 ± 0.1
*Enterococcus faecalis*	Roncal DOP	3LF6	0.3 ± 0.1
*Enterococcus faecalis*	Cabrales DOP	4LH12F	1.4 ± 0.2
*Enterococcus faecalis*	Cow’s milk curd	8ME4AF	0.4 ± 0.1
*Enterococcus faecalis*	Cow’s milk curd	8MD4F	0.3 ± 0.1
*Enterococcus faecalis*	Cow’s milk curd	8MH2F	0.2 ± 0.0
*Enterococcus faecium*	Terrincho DOP	2LC8	1.2 ± 0.2
*Enterococcus faecium*	Terrincho DOP	2LD4	1.2 ± 0.2
*Enterococcus faecium*	Terrincho DOP	2LF6	1.1 ± 0.2
*Enterococcus faecium*	Terrincho DOP	2LF9	0.9 ± 0.1
*Enterococcus faecium*	Cabrales DOP	4LA7F	1.7 ± 0.2
*Enterococcus faecium*	Cabrales DOP	4LF10F	1.4 ± 0.2
*Enterococcus faecium*	Cow’s milk curd	8MG2AF	0.2 ± 0.0
*Enterococcus faecium*	Cow’s milk curd	8MC4F	0.2 ± 0.1
*Enterococcus durans*	Terrincho DOP	2LA3A	1.2 ± 0.2
*Enterococcus durans*	Terrincho DOP	2LB3B	1.3 ± 0.3
*Enterococcus durans*	Terrincho DOP	2LD3	1.3 ± 0.3
*Enterococcus durans*	Terrincho DOP	2LD7A	1.3 ± 0.3
*Enterococcus durans*	Terrincho DOP	2LD7B	1.3 ± 0.3
*Enterococcus durans*	Terrincho DOP	2LG7A	1.4 ± 0.3
*Enterococcus durans*	Terrincho DOP	2LG12B	1.3 ± 0.3
*Enterococcus durans*	Cabrales DOP	4LA1F	1.7 ± 0.1
*Enterococcus durans*	Cabrales DOP	4LA4F	1.7 ± 0.3
*Enterococcus durans*	Cabrales DOP	4LB10F	1.7 ± 0.3
*Enterococcus durans*	Cabrales DOP	4LG3F	2.0 ± 0.1
*Enterococcus durans*	Cabrales DOP	4LG4F	1.7 ± 0.3
*Enterococcus durans*	Cabrales DOP	4LH11F	1.7 ± 0.1
*Leviactobacillus brevis*	Terrincho DOP	2MB9	0.9 ± 0.0
*Leviactobacillus brevis*	Terrincho DOP	2MH3	0.9 ± 0.1
*Leviactobacillus brevis*	Cabrales DOP	4ME9F	0.5 ± 0.1
*Leviactobacillus brevis*	Cabrales DOP	4MF9F	0.5 ± 0.1

DOP, Protected Designation of Origin.

**Table 5:** Technological characterization of PEA-producing *Enterococcus durans* isolates.

*E. durans* Isolate	Lactose Utilization (BCP Test)	Citrate Utilization	Proteolytic Activity	Milk Coagulating Capacity
4LA1F	+	−	High	Moderate
4LA4F	+	−	High	Null
4LB10F	+	−	High	Null
4LG3F	+	−	High	Null
4LG4F	+	−	High	Null
4LH11F	+	−	Low	Low
2LA3A	+	−	Null	Null
2LB3B	+	−	High	Null
2LD3	+	−	High	Null
2LD7A	+	−	Low	Null
2LD7B	+	−	Low	Null
2LG7A	+	−	Null	Null
2LG12B	+	−	High	Null
IPLA655	+	−	Low	Null

**Table 6:** Sugar and organic acid profiles of milk fermented with the different PEA-producing *Enterococcus durans* strains.

*E. durans* Isolate	Lactose	Glucose	Galactose	Citric Acid	Pyruvic Acid	Lactic Acid	Acetic Acid
4LA1F	387.1 ± 28.4	0	0.8 ± 0.05	0	0.5 ± 0.03	40.5 ± 2.3	9 ± 0.7
4LA4F	820.3 ± 67.7	0	2.3 ± 0.3	27.6 ± 5.5	0.3 ± 0.1	17.5 ± 1.9	2.6 ± 0.7
4LB10F	993.4 ± 72	0	2.4 ± 0.2	35.3 ± 2.3	0.2 ± 0.02	20 ± 1.9	2.4 ± 0.4
4LG3F	801.7 ± 92.2	0	1.9 ± 0.2	28.5 ± 3.4	0.1 ± 0.03	17.8 ± 1.7	2.1 ± 0.3
4LG4F	1048.1 ± 84.8	0	2.4 ± 0.2	37.6 ± 2.8	0.2 ± 0.03	20.6 ± 3.2	3 ± 0.5
4LH11F	946.5 ± 20.7	0	1.6 ± 0.06	35.6 ± 0.8	0.2 ± 0.009	35.2 ± 0.1	1.9 ± 0.03
2LA3A	821.2 ± 21.3	0	1.4 ± 0.08	30 ± 0.5	0.2 ± 0.05	22.9 ± 0.5	2.5 ± 0.5
2LB3B	913.6 ± 54.4	0	1.57 ± 0.2	33.7 ± 0.6	0.2 ± 0.06	27.8 ± 1.2	2.1 ± 0.4
2LD3	995.3 ± 80	0	1.4 ± 0.1	35.1 ± 0.4	0.2 ± 0.04	25.2 ± 2.8	3.4 ± 0.8
2LD7A	841.1 ± 14.1	0	1.6 ± 0.01	31.7 ± 0.9	0.15 ± 0.01	24.8 ± 1	2.4 ± 0.04
2LD7B	814.7 ± 63.3	0	1.1 ± 0.1	28.4 ± 0.1	0.1 ± 0.01	19.4 ± 0.7	2.2 ± 0.2
2LG7A	1112.5 ± 14.6	0	1.9 ± 0.2	41.2 ± 0.8	0.2 ± 0.01	17.6 ± 0.6	3.2 ± 0.2
2LG12B	815.9 ± 83.6	0	1.1 ± 0.1	27.9 ± 0.1	0.1 ± 0.05	15.5 ± 5.8	1.9 ± 0.7
IPLA655	1058.5 ± 97.7	0	1.6 ± 0.2	37 ± 0.02	0.1 ± 0.02	19.1 ± 1.7	2.2 ± 0.3
Milk	1075.8 ± 149.3	1.5 ± 0.2	2.71 ± 0.4	0	0	0	0

Results expressed as mg per 100 mL of milk.

**Table 7:** Normalized concentration, expressed as parts per thousands, of volatile compounds in milk fermented with the different PEA-producing *Enterococcus durans* isolates. The peak for each compound was normalized to the total area of the sample.

Compound	4LA1F	4LA4F	4LB10F	4LG3F	4LG4F	4LH11F	2LA3A	2LB3B	2LD3	2LD7A	2LD7B	2LG7A	2LG12B	IPLA655	MILK
**Aldehydes**															
Acetaldehyde	0.9 ± 0.1	1.5 ± 0.1	1.4 ± 0.03	1.6 ± 0.1	1.7 ± 0.1	1.7 ± 0.2	1.9 ± 0.4	2.1 ± 0.01	1.7 ± 0.4	1 ± 0.9	1.5 ± 1.3	2.9 ± 0.5	3.1 ± 0.2	3.1 ± 0.1	4.1 ± 0.5
Benzaldehyde	ND	ND	ND	ND	ND	ND	ND	ND	ND	ND	ND	ND	ND	ND	0.6 ± 0.5
**Ketones**															
2-Tridecanone	1.1 ± 0.1	1.7 ± 0.1	1.4 ± 0.2	1.3 ± 0.1	1.3 ± 0.1	1.1 ± 0.1	1 ± 0.04	1 ± 0.2	0.9 ± 0.02	1 ± 0.07	1 ± 0.2	1.1 ± 0.1	1.4 ± 0.1	1.4 ± 0.03	1.6 ± 0.1
2-Pentadecanone	0.4 ± 0.05	ND	ND	ND	ND	ND	ND	ND	ND	ND	ND	ND	ND	ND	0.6 ± 0.02
2-Heptanone	1.1 ± 0.2	2.5 ± 0.4	2.7 ± 0.6	2.9 ± 0.4	3.2 ± 0.3	3 ± 0.1	3.1 ± 0.5	2.7 ± 0.03	3 ± 0.5	3.3 ± 0.1	5.3 ± 3.8	5.5 ± 6.1	10.4 ± 0.7	10.2 ± 0.6	14.7 ± 0.6
2-Nonanone	4.3 ± 0.3	8.1 ± 0.5	7.4 ± 0.6	7.9 ± 0.2	7.7 ± 0.1	8.5 ± 0.4	8.3 ± 1.6	8.3 ± 0.4	7.9 ± 0.3	8 ± 0.3	7.9 ± 0.1	7.1 ± 0.6	8.1 ± 0.3	8 ± 0.6	9.5 ± 1.3
2-Undecanone	3.2 ± 0.2	4.4 ± 0.1	3.9 ± 0.4	3.9 ± 0.2	3.4 ± 0.2	3.8 ± 0.1	3.7 ± 0.6	3.7 ± 0.4	3.4 ± 0.1	3.6 ± 0.2	3.6 ± 0.3	3 ± 0.2	4.2 ± 0.2	4 ± 0.1	3.7 ± 0.1
**Lactones**															
Acetoin	21.2 ± 3.2	ND	12.5 ± 13.5	ND	ND	2.6 ± 2.3	ND	4.1 ± 0.3	1.4 ± 1.4	2.2 ± 0.8	ND	9.7 ± 0.5	ND	ND	ND
γ-Dodecalactone	1.2 ± 0.05	1.9 ± 0.1	1.9 ± 0.3	1.9 ± 0.1	1.9 ± 0.2	1.7 ± 0.1	1.8 ± 0.1	1.7 ± 0.2	1.7 ± 0.03	1.8 ± 0.1	1.6 ± 0.02	1.8 ± 0.1	1.9 ± 0.2	1.9 ± 0.2	1.9 ± 0.01
δ-Dodecalactone	1.2 ± 0.02	1.6 ± 0.05	1.6 ± 0.1	1.7 ± 0.1	1.7 ± 0.2	1.6 ± 0.2	1.5 ± 0.1	1.4 ± 0.1	1.4 ± 0.06	ND	1 ± 0.9	1.5 ± 0.1	1.7 ± 0.2	1.6 ± 0.2	1.6 ± 0.1
δ-Decalactone	1.2 ± 0.04	1.6 ± 0.1	1.5 ± 0.2	1.6 ± 0.1	1.6 ± 0.1	1.4 ± 0.1	1.4 ± 0.1	1.5 ± 0.2	1.4 ± 0.03	1.4 ± 0.1	1.4 ± 0.1	1.3 ± 0.1	1.5 ± 0.05	1.5 ± 0.1	1.3 ± 0.03
**Acids**															
Acetic acid	96 ± 11.4	37.8 ± 1.6	45.7 ± 7.1	32.2 ± 3.4	29.6 ± 0.8	28.8 ± 2.7	31.9 ± 2.5	29.8 ± 4.2	28.3 ± 2.3	26.2 ± 4.8	22 ± 4.7	18.8 ± 2.6	13.8 ± 1.9	14.9 ± 2.2	ND
Butanoic acid	6 ± 0.6	8 ± 0.3	6.5 ± 1	6.9 ± 0.3	6.5 ± 0.1	7.9 ± 0.5	6.8 ± 1.5	6.7 ± 0.2	6.4 ± 0.5	5.9 ± 0.2	6.1 ± 0.6	4 ± 0.2	6.2 ± 0.4	5.9 ± 0.3	1.3 ± 1.1
Benzoic acid	4.3 ± 3.7	ND	ND	ND	ND	3.2 ± 2.8	ND	ND	ND	ND	ND	ND	ND	ND	ND
Hexanoic acid	58.8 ± 3	59.6 ± 3.3	60 ± 2.3	60.4 ± 7	60.6 ± 1.6	75.6 ± 3.7	66.6 ± 13.9	70 ± 1.6	67.2 ± 4.2	65.9 ± 2.4	65.1 ± 4.4	36.1 ± 1.7	65.3 ± 3.9	64.3 ± 2.4	5.8 ± 0.7
Heptanoic acid	1 ± 0.3	1 ± 0.1	ND	ND	ND	ND	ND	ND	ND	ND	0.5 ± 0.5	ND	1 ± 0.04	1 ± 0.1	ND
Octanoic acid	81.9 ± 2.9	97.6 ± 4.5	97.9 ± 7.5	101.4 ± 11.3	102.1 ± 3	110.5 ± 3.6	104 ± 18	108.2 ± 9.1	103.8 ± 4.7	104.4 ± 6.8	104.9 ± 5.3	66.6 ± 4.7	105.8 ± 4.6	105.1 ± 3.5	9.1 ± 1.9
Nonanoic acid	1.6 ± 0.3	2.1 ± 0.5	1.7 ± 0.3	2.1 ± 0.5	1.6 ± 0.1	1.7 ± 0.1	1.8 ± 0.5	1.7 ± 0.9	1.3 ± 0.2	1 ± 0.2	1.4 ± 0.06	2.2 ± 1.3	1.7 ± 0.5	1.8 ± 0.3	ND
n-Decanoic acid	42 ± 2	59 ± 4.2	58.5 ± 6	54.1 ± 1.7	56 ± 2.4	58.5 ± 0.7	56.9 ± 9.8	54 ± 2.2	53.9 ± 2.5	62.8 ± 6.8	61.5 ± 4.2	50.5 ± 1.6	62.4 ± 1.5	58.3 ± 2	9.9 ± 3
9-Decenoic acid	4.3 ± 0.1	5.4 ± 0.6	5.1 ± 0.6	4.1 ± 0.4	4.4 ± 0.2	5.3 ± 0.2	4.6 ± 1.1	4.6 ± 0.7	4.5 ± 0.3	5.5 ± 0.4	5.6 ± 0.9	3.2 ± 0.5	5.8 ± 0.3	5.3 ± 0.3	ND
Dodecanoic acid	4.3 ± 0.1	6.6 ± 0.3	7 ± 1	5.8 ± 1.6	6.2 ± 0.5	6.9 ± 0.3	6.2 ± 1.5	4.7 ± 0.1	4.2 ± 0.3	8.3 ± 1.5	7.1 ± 2.6	ND	6.5 ± 0.9	5.9 ± 1.2	ND
9-Octadecenoic acid	ND	ND	ND	ND	ND	ND	ND	ND	ND	ND	1.4 ± 1.2	ND	ND	ND	ND
Cyclohexanecarboxylic acid	1.5 ± 0.2	ND	ND	ND	ND	2.2 ± 0.9	6.7 ± 2.9	7.7 ± 3	9 ± 0.6	10.6 ± 0.8	9.5 ± 1.2	ND	11.6 ± 2	14.6 ± 0.9	ND
**Alcohols**															
2-Nonanol	1 ± 0.1	ND	0.6 ± 0.04	ND	ND	ND	ND	ND	ND	ND	ND	ND	ND	ND	ND
2-methyl-1-Hexadecanol	1 ± 0.1	ND	ND	ND	ND	ND	ND	ND	ND	ND	ND	ND	ND	ND	ND
**Sulphur compounds**															
Dimethyl sulphone	ND	ND	0.7 ± 0.1	ND	ND	ND	ND	ND	ND	ND	0.4 ± 0.4	1.2 ± 0.3	ND	0.4 ± 0.3	1.7 ± 0.3

ND: Not Detected.

**Table 8:** Antimicrobial susceptibility profiles of the PEA-producing *Enterococcus durans* strains.

*E. durans* Isolate	AMP10	CN10	K30	S10	C30	TE30	VA30	E15	DA2
4LA1F	S	S	S	S	S	S	S	S	S
4LA4F	S	S	S	S	S	S	S	S	R
4LB10F	S	S	S	R	S	S	S	S	R
4LG3F	S	S	S	R	S	S	S	R	R
4LG4F	S	S	S	S	S	S	S	R	R
4LH11F	S	S	S	S	S	S	S	S	S
2LA3A	S	S	S	S	S	S	S	S	S
2LB3B	S	S	S	S	S	S	S	S	S
2LD3	S	S	S	S	S	S	S	S	S
2LD7A	S	S	S	S	S	S	S	S	S
2LD7B	S	S	S	S	S	S	S	S	S
2LG7A	S	S	S	S	S	S	S	S	S
2LG12B	S	S	S	R	S	S	S	S	R
IPLA655	S	S	S	R	S	S	S	S	R

AMP, Ampicillin; CN, Gentamicin; K, Kanamycin; S, Streptomycin; C, Chloramphenicol; TE, Tetracyclin; VA, Vancomycin; E, Erythromycin; DA, Clindamycin.

**Table 9:** Minimum inhibitory concentration (MIC) of ampicillin for PEA-producing *Enterococcus durans* isolates.

*E. durans* Isolate	MIC (mg/L)
4LA1F	0.5
4LA4F	0.25
4LB10F	0.25
4LG3F	0.25
4LG4F	0.25
4LH11F	1
2LA3A	1
2LB3B	0.5
2LD3	1
2LD7A	1
2LD7B	1
2LG7A	1
2LG12B	1
IPLA655	0.5

**Table 10:** Virulence gene screening of PEA-producing *Enterococcus durans* isolates.

*E. durans* Isolate	*gelE*	*efaA*	*esp*	*hylEfam*	*IS16*
4LA1F	-	-	-	-	-
4LA4F	-	-	-	-	-
4LB10F	-	-	-	-	-
4LG3F	-	-	-	-	-
4LG4F	-	-	-	-	-
4LH11F	-	-	-	-	-
2LA3A	-	-	-	-	-
2LB3B	-	-	-	-	-
2LD3	-	-	-	-	-
2LD7A	-	-	+	-	-
2LD7B	-	-	-	-	-
2LG7A	-	-	-	-	-
2LG12B	-	-	-	-	-
IPLA655	-	-	-	-	-

**Table 11:** Biogenic amine production in cultures of PEA-producing *Enterococcus durans* isolates.

*E. durans* Isolate	Tyramine	Histamine	Putrescine (AGDI)	Putrescine (ODC)
4LA1F	**+**	-	-	-
4LA4F	**+**	-	-	-
4LB10F	**+**	-	-	-
4LG3F	**+**	-	-	-
4LG4F	**+**	-	-	-
4LH11F	**+**	-	-	-
2LA3A	**+**	-	-	-
2LB3B	**+**	-	-	-
2LD3	**+**	-	-	-
2LD7A	**+**	-	-	-
2LD7B	**+**	-	-	-
2LG7A	**+**	-	-	-
2LG12B	**+**	-	-	-
IPLA655	**+**	-	-	-

AGDI, agmatine deiminase pathway; ODC, ornithine decarboxylase pathway.

**Table 12:** Production of tyramine and β-phenylethylamine by strain *E. durans* IPLA655 and the mutant *Δtdc* after 48 h of incubation in GM17 medium supplemented with 2 mM tyrosine and phenylalanine.

Strain	Tyramine (mM)	PEA (mM)
IPLA655	2.5	2.1
IPLA655-*Δtdc*	ND	ND

## Data Availability

The dataset is available on request from the authors.

## Supplementary Materials

The following supporting information can be downloaded at: https://www.mdpi.com/article/10.3390/microorganisms13050966/s1, Figure S1: Proteolytic activity of the isolates examined using PCA plate supplemented with 2% UHT semi-skimmed cow’s milk. Figure S2: Phylogenetic tree of amino acidic sequences of tyrosine decarboxylases (TDC) from different species of the Lactic Acid Bacteria group.
