# Supplementary material for: Isolation and Characterization of β-Phenylethylamine-Producing Lactic Acid Bacteria from Dairy Products
Source: Microorganisms. 2025 Apr 23;13(5):966. doi: 10.3390/microorganisms13050966 (PMC12114284; doi:10.3390/microorganisms13050966)
Supplement: Supplementary file 1 [file microorganisms-13-00966-s001.zip › microorganisms-3538840-supplementary.pdf]

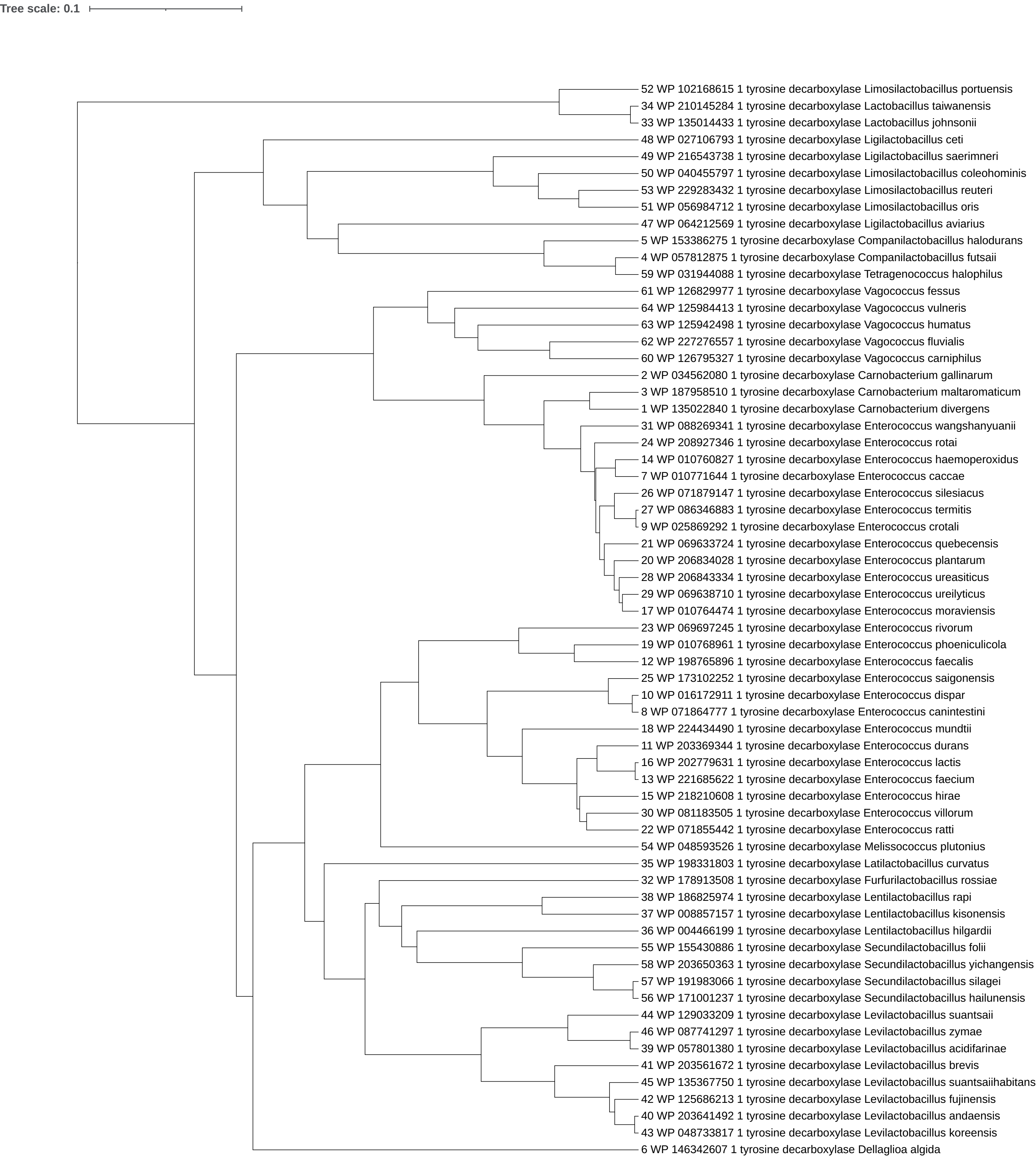

Figure S2: Phylogenetic tree of amino acidic sequences of tyrosine decarboxylases (TDC) from different species of the Lactic Acid Bacteria group. The tree was generated by using the unweighted pair group method with arithmetic means (UPGMA) and employing Clustal Omega software (https://www.ebi.ac.uk/jdispatcher/msa/clustalo). The generated phylogenetic tree was visualized using the iTOL web server (https://itol.embl.de/).

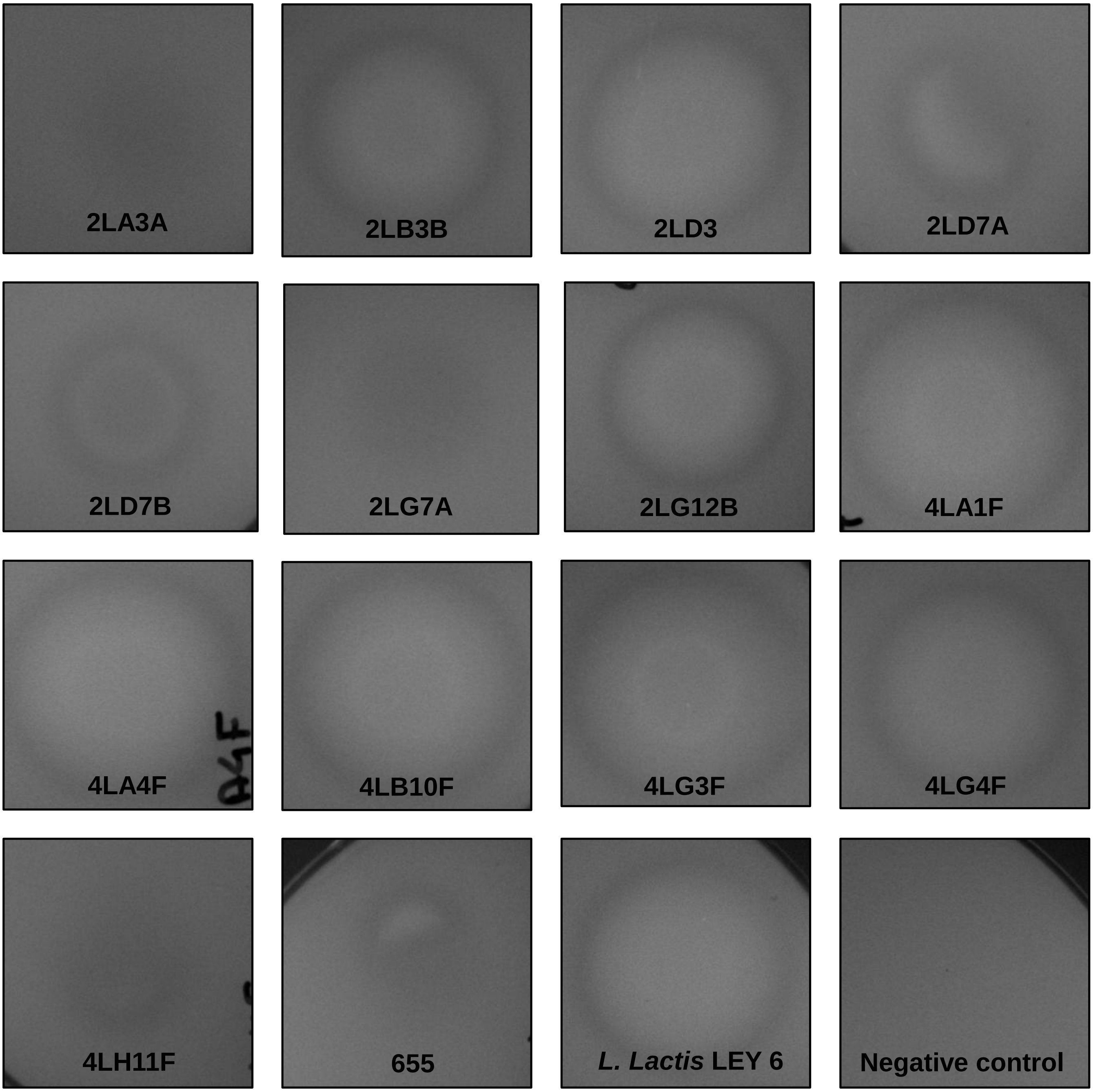

Figure S1: Proteolytic activity of the isolates examined using PCA plates supplemented with 2% UHT semi-skimmed cow's milk (Oxoid). Isolated cultures were spotted onto these plates and incubated at 32°C for 48 h. A clear zone around the colonies indicated proteolytic activity. *L. lactis* LEY6 was used as a positive control.
